# Sick Day Management Plans for Aboriginal and/or Torres Strait Islander Peoples With Chronic Kidney Disease on the Cape York Peninsula of Australia: Health Workers' Perspectives

**DOI:** 10.1111/ajr.13223

**Published:** 2025-01-25

**Authors:** Luke Calleja, Beverley Glass, Selina Taylor, Kisha Neville, Leanne Brown, Andrea Miller, Alice Cairns

**Affiliations:** ^1^ College of Medicine and Dentistry James Cook University Townsville Queensland Australia; ^2^ Townsville Hospital and Health Service Townsville Queensland Australia; ^3^ Murtupuni Centre for Rural and Remote Health James Cook University Townsville Queensland Australia; ^4^ Torres and Cape Hospital and Health Service Weipa Queensland Australia; ^5^ School of Nursing and Midwifery Griffith University Brisbane Queensland Australia; ^6^ Australian Institute of Tropical Health and Medicine James Cook University Townsville Queensland Australia

**Keywords:** acute kidney injury, Cape York Peninsula, chronic kidney disease, dehydration, nephrotoxicity, sick day management plans

## Abstract

**Objective:**

This study aimed to explore the perspectives of healthcare professionals on the utility of sick day management plans for people with chronic kidney disease (CKD) in remote communities and collaboratively design a sick day management plan resource.

**Design:**

This qualitative study utilised two phases of data collection: preliminary observational data and semi‐structured interviews. The research design and analysis were guided by the normalisation process theory (NPT) framework, tailored for complex interventions in healthcare.

**Setting:**

Three First Nations communities and one remote mining community in Cape York, Australia.

**Participants:**

In‐person semi‐structured interviews were conducted with 23 primary healthcare workers; 40% identified as Aboriginal and/or Torres Strait Islander.

**Results:**

The study identified three themes relating to feasibility of implementation: (1) resource coherence and readability, (2) suitability for integration into the care model and (3) safety and risk associated with sick day management plans. Iterative development of resources followed, incorporating feedback from the participants. Recommendations emerged for enhanced readability and coherence, including further co‐design with individual communities and consumers, content simplification, incorporation of Aboriginal and/or Torres Strait Islander artwork and language and a flow chart structure.

**Conclusion:**

The study underscores the importance of culturally sensitive resource design and the active involvement of Aboriginal and/or Torres Strait Islander communities in healthcare improvement. Future research should explore cost‐effective methods for personalised sick day management plans.

**Summary**

- What is already known on this subject?
  ○ Polypharmacy is common in people with CKD, who are prescribed medications with nephrotoxic potential (SADMANS) which, if the person is dehydrated, present a risk of acute kidney injury.
  ○ Sick day management plans may guide people with CKD to temporarily discontinue these SADMANS medications if they are dehydrated.
  ○ Although CKD is a serious and increasingly common health problem in Aboriginal and/or Torres Strait Islander populations, the application of sick day management plans to this population remains unexplored.
- What does this study add?
  ○ For sick day management plans to be successfully implemented in remote Aboriginal and/or Torres Strait Islander communities, investment in an ‘easy to personalise’ educational tool will be required. This would most likely be a technology‐based solution.
  ○ Healthcare workers recognised the potential of sick day management plans as a valuable tool for patient education and management, addressing both CKD in general and specifically during sick days.
  ○ Collaboratively designing educational resources with First Nations staff in remote primary health clinics was a valued process, producing real‐time opportunities for providing education on CKD management and context‐specific education development.

## 1 Introduction

Aboriginal and/or Torres Strait Islander people accounted for approximately 3% of the Australian population in 2018/2019, but represented 21% of newly diagnosed people with chronic kidney disease (CKD) [1, 2]. The prevalence of end‐stage kidney disease is 13.6 times higher in this population compared to non‐Indigenous Australians, resulting in higher morbidity and mortality associated with CKD [2]. The aetiology of CKD is complex and multifactorial and involves various risk factors including social determinates of health, poor nutrition, limited access to healthcare services, reduced kidney reserve at birth and cultural and language barriers in the treatment and management [3, 4]. The prevalence of risk factors for CKD including diabetes and hypertension is also higher in Aboriginal and Torres Strait populations comparted non‐Indigenous Australians, type two diabetes, for example, is 2.8 times more prevalent [5]. CKD is a significant public health concern on the Cape York Peninsula (the Cape), far north Queensland, where Aboriginal and/or Torres Strait Islander people make up 46% [6] of the population, experiencing a higher impact of CKD than their non‐Indigenous counterparts [2]. To address the unique health and service delivery needs in the Cape, the Cape York Kidney Care Team delivers specialist care on a fly‐in, fly‐out basis, operating within primary care settings across the remote communities [7]. Language and communication pose a challenge in healthcare delivery on the Cape, as many people speak in traditional languages such as Wik and Creole [8].

People with CKD are prescribed a range of medications to control blood pressure, reduce proteinuria, prevent cardiovascular complications and delay the progression of kidney disease [9, 10]. The average range of medications prescribed for people with CKD is between 4.1 and 16.2 [10]. Medications with nephrotoxic potential, collectively known as SADMANS (sulfonylureas, angiotensin‐converting enzyme inhibitors, diuretics, metformin [5], angiotensin‐2 receptor antagonists, non‐steroidal anti‐inflammatories and sodium‐glucose co‐transporter‐2 (SGLT2) inhibitors) [11], are commonly prescribed for CKD in the Cape [11, 12], presenting a risk of acute kidney injury (AKI) if taken while dehydrated. Recognising these risks, there is potential for implementing sick day management plans to guide patients on temporarily discontinuing SADMANS medications during dehydration episodes [11, 13].

Although the principle of a sick day management plan has been informally tested in metropolitan areas of Australia, its application in Aboriginal and/or Torres Strait Islander populations remains unexplored [11]. This study aims to evaluate healthcare workers' perspectives on sick day management plans for people with CKD in the Cape. Additionally, the study aims to collaboratively design a resource that aligns with the unique needs of the community.

## 2 Methods

### 2.1 Study Design

This qualitative study employed two concurrent phases of data collection: preliminary observational data collection and semi‐structured interviews. The study design and analysis were guided by the Normalisation Process Theory (NPT) framework, which is tailored for developing, implementing and evaluating complex interventions in healthcare settings [14]. The adapted NPT framework in this study incorporated the components of intervention context, coherence, cognitive engagement, collective action and reflexive monitoring [14].

Ethics for this study were approved by the Far North Queensland Human Research Ethics Committee (HREC/2021/QCH/72719 (May ver2) – 1519).

### 2.2 Setting

This study was conducted in the Cape York Peninsula of Queensland, located in Northern Australia [15], in four communities with a total population of 6516 [16]. All four communities are classified as very remote (MM7) [17]; one is an Aboriginal community, two identify as Aboriginal and/or Torres Strait Islander communities and one is a mining town that provides ‘hub’ services for the other communities. The Cape spans 137 000 km^2^ and faces considerable geographical isolation from its nearest regional centre and specialist nephrology clinic, Cairns, Queensland [15, 18].

### 2.3 Procedure

Over 2 weeks in June 2022, contextual, demographic and environmental data were collected by observing the Cape York Kidney Care Team's outreach clinics in the four communities. Field notes were taken to document patterns related to patient engagement, resources and care practices. The observations informed the development of interview questions and an initial sick day management plan resource. The interview questions were piloted with a local Aboriginal Health Worker and were refined to align with components of an adapted version of the Normalisation Process Theory (NPT) framework [14].

### 2.4 Recruitment

Approximately 45 healthcare workers from six Cape primary healthcare clinics, and the Cape York Kidney Care team were invited to participate in semi‐structured interviews. Invitations were extended through email, telephone and/or in‐person by members of the research team. Upon expressing their interest in participation, individuals were then provided with an Information Sheet and given an explanation of the study details before informed consent was obtained. Interviews were conducted over a 3‐week period in January and February 2023 at community health clinics. Interviews were audio‐recorded and lasted between 20 and 60 min duration. All participants were aware that the primary researcher (Author 1) was a health student completing a research honours degree; the student had been on placement previously in the communities and had a strong interest in rural and Aboriginal and/or Torres Strait Islander health. Participants were made aware that the senior researcher supporting data collection (Author 7), who was also present during interviews, was supporting a larger evaluation of the Cape York Kidney Care service.

Participants were asked a variety of questions aimed at comprehensively exploring various aspects of managing medicines during sick days within the context of CKD care. Although the specific questions varied across interviews, a set of common inquiries was shared, including the following:

1. What are your current recommendations for patients on managing their medicines during sick days, and how is that advice delivered?
2. Can you identify any potential barriers to patient‐initiated exclusion of SADMANS medicines during sick days?
3. Do you believe providing patients with sick day education tools to help them guide their own medication management would be effective and safe? Why or why not?

Further to participant responses, additional questions were asked to clarify perspectives and extract deeper insights. This iterative approach formed the foundation for gathering comprehensive data which allowed for a more nuanced understanding and enriched the dataset.

During the interview, participants were presented with an initial example of a sick day management plan and were provided with an explanation of its purpose. Feedback was then sought through open‐ended questions, including (Appendix A: Interview Schedule in Data S1):

1. What are your initial thoughts on seeing this?
2. What would you consider before handing this to a patient?
3. Are there any components that stand out to you as being beneficial or potentially challenging for a patient?

**FIGURE 1:**
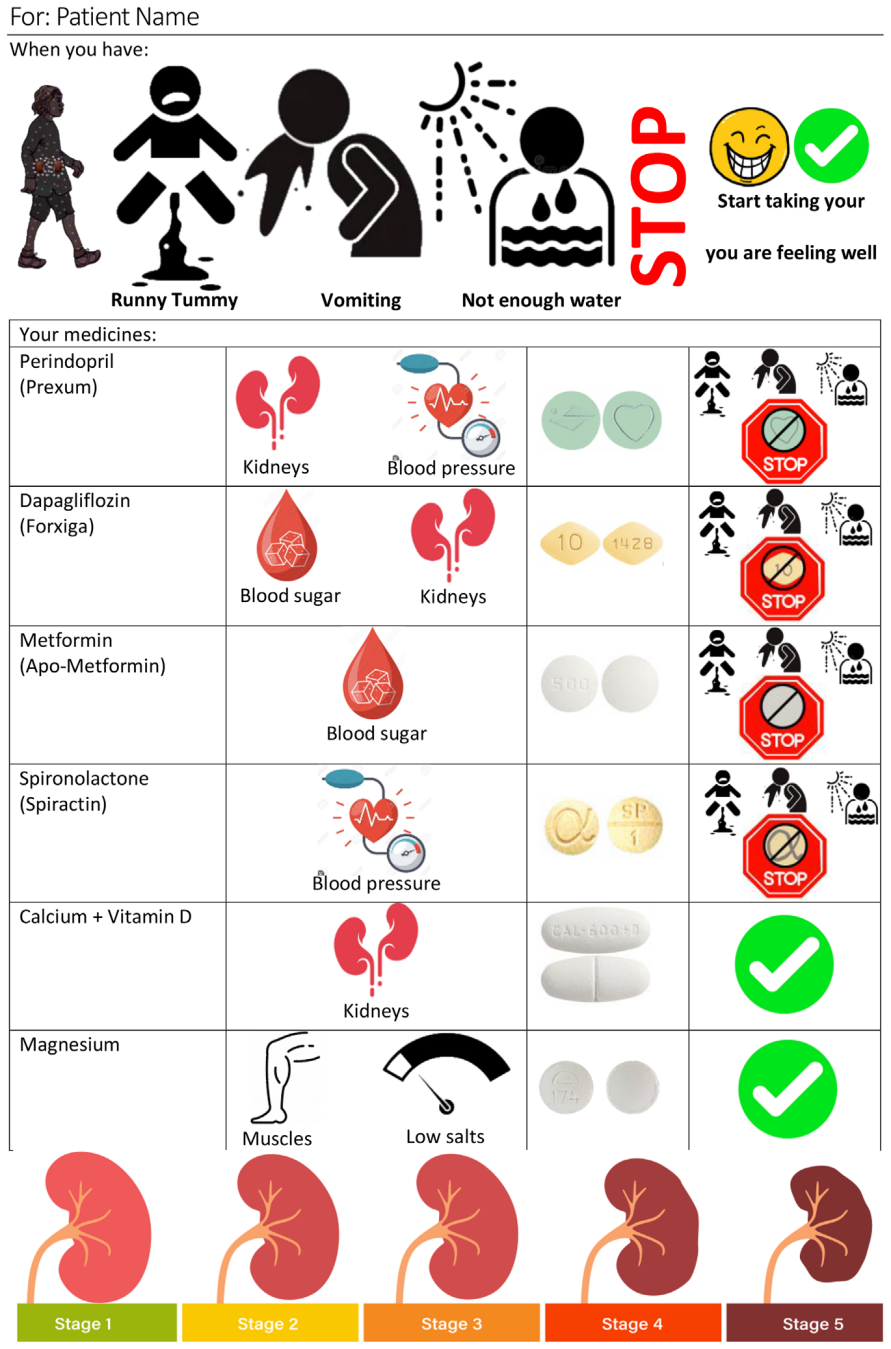
Final proposed sick day management plan resource.

After blocks of three to four interviews, based on the feedback received, the example sick day management plan was adjusted. When there was no new feedback, a final draft sick day management plan was developed (Figure 1).

Interviews were transcribed verbatim, and the transcriptions were manually checked for reliability, allowing for the recognition of subtle participant cues to be noted. Two researchers (Author 1 and Author 7) conducted all interviews. To enhance reflexivity, Authors 1 (male) and 7 (female) engaged in debriefing sessions after each interview, making post‐interview notes on emerging themes. Both authors involved in the data collection and analysis process are non‐Indigenous allied health clinicians with expertise or experience in rural and remote service delivery. Author 7 had lived in the region previously and has expertise in qualitative research methodologies and research with Aboriginal and/or Torres Strait Island peoples. Member checking was not conducted, at the preference of participants and because of high staff attrition rates.

### 2.5 Data Analysis

Following the proofreading and confirmation of each transcript's accuracy, transcriptions managed using Nvivo 12 and thematic analysis was conducted using Braun and Clark's method [19]. This involved author one rereading transcripts and generating initial codes (inductive coding) before deductively coding against the constructs and components of the NPT analysis framework (context, cognitive participation, collective action and coherence) [14, 19, 20]. Data inductively coded were then refined into overarching themes that considered the feasibility of integrating a sick day management plan into kidney care on the Cape. Themes were then rigorously reviewed by Authors one and two for coherence and relevance and discussed with Authors three and seven. In addition to exploring health workers perspectives of the sick day management plan, a ‘roadmap’ was developed which displayed the iterations of the sick day management plan, outlining the feedback and adjustments that were made throughout the 2‐week interview process.

## 3 Results

A total of 21 interviews were conducted, involving 23 participants across 10 different health disciplines. Participants included administrative staff (*n* = 2, 9%), Aboriginal and/or Torres Strait Islander Health Workers or Practitioners (*n* = 8, 36%), Registered Nurses (*n* = 5, 22%), Nurse Practitioners (*n* = 2, 9%), General Practitioners (*n* = 1, 4%) and Allied Health staff (*n* = 3, 13%) (Table 1).

**TABLE 1:** Participant characteristics (*n* = 23).

Gender	Male (17%, *n* = 4)	Female (83%, *n* = 19)	Diverse (*n* = 0)
Identifies as Aboriginal and Torres Strait Islander	Yes (39%, *n* = 9)	No (60%, *n* = 14)	
Time spent in community	< 12 months = (21%, *n* = 5)	12–24 months (26%, *n* = 6)	> 24 months (52%, *n* = 12)
Fly‐in, fly‐out	Yes (48%, *n* = 11)	No (52%, *n* = 12)	

Exploring the overall feasibility of sick day medicines management, our study identified three primary themes: resource coherence and readability, resource integration into the model of care and resource safety and risk.

### 3.1 Resource Coherence and Readability

Examination of healthcare staff's ability to deliver education to people with complex chronic kidney disease revealed challenges in clarity relating to components of the NPT Framework, including context, coherence, cognitive participation and reflexive monitoring. Aboriginal and/or Torres Strait Islander health workers expressed concerns about the adequacy of resources, citing difficulties in patient understanding. The clarity and coherence of a sick day management plan were acknowledged as crucial, with participants suggesting that images and native language enhance comprehension.

### 3.2 Resource Integration Into the Model of Care

Cultural context of care provision in certain communities was highlighted as a potential barrier to the integration of sick day management plans relating to the NPT Framework components of context, cognitive participation and collective action of the resource. Participants noted a prevailing health service focus on acute conditions rather than chronic care management. The feasibility of integrating the resource into routine community clinic consults emerged as a key consideration, with suggestions that healthcare workers, particularly Aboriginal and/or Torres Strait Islander health workers, could play a pivotal role in incorporating these resources into consultations.

### 3.3 Resource Safety and Risk

Concerns were raised regarding the safety and risks associated with the complexity of medication regimens for people with chronic conditions relating to NPT components of resource context, coherence, cognitive participation and collective action. Participants emphasised the challenge of identifying specific medicines for exclusion in pre‐packaged dosage administration aids during periods of illness, posing potential risks. Participants also expressed concerns about overwhelming people with multiple images in the sick day management plan, potentially leading to disengagement and increased risks.

The interview data were coded against the components of the NPT (Table 2).

**TABLE 2:** Five components from the Normalisation Process Theory (NPT) framework and a description of their application in this study.

Constructs	Description	Components
Context	Pre‐existing context in which the sick day management plan resource will be implemented	Barriers
		Facilitators
		Cultural norms
		Adherence
Coherence	Clarity or intelligibility of the sick day management plan resource for use with chronic kidney disease patients on the Cape	Communal specification
		Individual specification
		Internalisation
		Differentiation
Cognitive participation	Stakeholders' engagement, understanding and commitment to integrate sick day management plans within existing routines	Initiation
		Enrolment
		Legitimation
		Activation
Collective action	Operational work required to properly utilise the resource including steps for integration into the model of care	Interactional workability
		Relational integration
		Skillset workability
		Contextual integration
Reflexive monitoring	Appraisal of the resource by means of an iterative feedback process	Systemisation
		Communal appraisal
		Individual appraisal
		Reconfiguration

### 3.4 Context

Within the pre‐existing context of implementing the sick day management plan, several subthemes emerged, highlighting both barriers and facilitators. Cultural norms, adherence and the influence of family structures played pivotal roles in shaping the landscape of medication management for people with complex CKD.

Aboriginal and/or Torres Strait Islander health workers highlighted a significant challenge in delivering comprehensive education and management to patients, and the perceived inadequacy of skills and available resources, hindering the provision of counselling for people with complex needs. An Aboriginal and/or Torres Strait Islander staff member highlighted this concern, stating,

Some of the resources aren't, they're not to a level where people can understand. Like what I can use for myself, for example. (P17)

Perceived service delivery models within some Cape York communities posed an additional barrier. The reluctance to embrace sick day management plans stemmed from a perception that clinics primarily focus on acute conditions rather than chronic care management.

It's not part of the culture, I suppose, to provide that service, not part of the service mentality. (non‐Indigenous participant, P19)

Adherence to prescribed regimens was identified as a facilitator, with the family structure and attitudes significantly influencing people's adherence. Participants acknowledged that patients with supportive and ‘healthcare positive’ families were more likely to adhere to their medicines, regardless of their apparent health.

It's usually the family that pushes our clients. (Aboriginal and/or Torres Strait Islander participant, P7)

The influence of cultural norms emerged as a key subtheme, shaping perceptions and practices related to healthcare provision. Cultural reluctance within specific communities contributed to a hesitancy in adopting sick day management plans, reflecting a broader perspective that clinics primarily address acute conditions rather than chronic care management.

### 3.5 Resource Coherence

Participants across both Aboriginal and/or Torres Strait Islander and non‐Indigenous healthcare workers unanimously agreed on the potential benefits of a sick day management plan. The sick day management plan iterations were consistently described as clear and succinct. The integration of images and native language was highlighted for its role in enhancing both patient and staff understanding, with particular emphasis from Aboriginal and/or Torres Strait Islander health workers on the effectiveness of pictorial explanations for medication identification.

Communal aspects were underscored by the belief that frequent patient education could establish a connection between patients and healthcare teams. This connection, participants noted, is pivotal for facilitating engagement and ensuring the retention of essential information, enhancing the feasibility of improving patient outcomes.

Diverse perspectives emerged regarding the use of multiple images in the sick day management plan. While Aboriginal and/or Torres Strait Islander health workers found value in patients identifying tablets based on use and appearance rather than generic or brand names, non‐Indigenous staff expressed reservations. Concerns were voiced that the use of multiple images might overwhelm or confuse patients, especially those with a limited understanding of their condition and treatments.

Participants consistently highlighted the challenge faced by people with chronic conditions who contend with many different medicines in their treatment regimens. The complexity arising from this diversity poses a considerable obstacle when attempting to identify specific medicines for exclusion in dosage administration aids, particularly during vulnerable periods of illness.

Eight tablets in one blister, so it's hard to tell them apart, and a lot of them are white. So, you can't say don't take these little white ones take this big one only. (non‐Indigenous participants, P19)

### 3.6 Cognitive Participation

The network of care between the primary healthcare teams and the specialist kidney team was reported as vital for the sustainability of sick day management plans. The primary health staff discussed the importance of education and training between the Cape York Kidney Care team to build knowledge and participation in supporting sick day management.

Knowing what you're taking, why, and how it affects your body is essential. In‐services and education on medications are important. (Aboriginal and/or Torres Strait Islander participant, P3)

Patient cognitive engagement with the resource consistently correlated with an understanding of their medical condition, medication regimen and the importance of adhering to the sick day management plan to prevent acute kidney injury. Aboriginal and/or Torres Strait Islander health workers universally believed that most people with CKD are eager to learn about their condition, extending beyond medication use to include how medications are made—a reflection of the region's strong traditional medicine culture.

They want to know, how the medicines are helping the body? How did they make it? Where do you get them? How is it made in a factory? What are the ingredients in it? Yes, that is very, very important. (Aboriginal and/or Torres Strait Islander participant, P1)

While Aboriginal and/or Torres Strait Islander health workers stressed the significance of patient engagement, acknowledging the patients' curiosity about the origin and composition of medications, some non‐Indigenous participants disagreed. They perceived a lack of interest among people with CKD, attributing it to the ‘invisible’ nature of kidney disease, with heightened concern only emerging at advanced stages threatening dialysis.

Consensus among all participants was the necessity of frequent education, with a focus on utilising available resources within communities rather than relying on periodic visits from renal specialist outreach teams. Aboriginal and/or Torres Strait Islander health workers advocated for the regular use of a sick day management plan to reinforce education on medicine management and enhance ongoing cognitive engagement.

### 3.7 Collective Action

Clinical management staff proposed integrating the sick day management plan into routine community clinic consultations, suggesting that Aboriginal and/or Torres Strait Islander health workers incorporate it during patient interactions. Some nursing staff recommended clinic visits during illness for reviewing and updating sick day management plans to address potential issues related to low health literacy when patients interpret them at home.

However, skepticism arose about the current knowledge levels of community staff to effectively counsel patients on sick day management plans, leading to suggestions for additional staff training. The simplicity of the resource was acknowledged, with non‐clinical staff potentially playing a role in patient education. Concerns about patients' ability to retain information prompted the idea of repeated education by healthcare professionals using the resource. Clinical staff collectively endorsed clinic visits for healthcare worker–guided interpretation of sick day management plans, prioritising patient safety.

There's no education for the workers about why patients are on their medicines or that when patients are on those medicines and are unwell, they need to stop them and call their doctor. (Aboriginal and/or Torres Strait Islander participant, P14)

Doctors, nurses and allied health staff emphasised clinic presentations for patient education, documentation and continuity of care, envisioning a sick day management resource being a positive addition to healthcare workers' educational toolkits.

Concerns were raised, however, about the time‐intensive nature of creating individualised sick day management plans, including the need for updated medication images. Participants suggested the development of templates for efficiency, but highlighted challenges in maintaining accuracy due to frequent changes in patient medications.

The issue is whether or not all the medications look the same. Because the brand of the tablet we use changes probably monthly sometimes. (Non‐Indigenous participant, P8)

The consensus among participants was that while the sick day management plans could enhance patient education, the labour‐intensive nature of customisation poses practical challenges that need to be addressed for successful implementation.

### 3.8 Reflexive Monitoring

Reflexive monitoring assessed participants' responses to the evolving sick day management plan resource during the 2‐week interview period. Recommendations for refining the resource aimed at improving readability and emphasising key aspects. Enlarging symptoms related to dehydration was suggested for clarity, along with listing medications by both generic and brand names to accommodate patient recognition. Including details on how long patients should pause their medicines and providing information on the decline of kidney function were considered essential for patient understanding.

I think the education is very important with the pictures that are on the resource… We don't see our body on the inside like this … And kids would love to see the differences in healthy vs unhealthy bodies. (Aboriginal and/or Torres Strait Islander participant, P1)

Participants appreciated the educational aspect of the resource, particularly the visual representation of internal body structures, believed to be beneficial for patient comprehension. After adjustments to Version 2 of the sick day management plan (Data S1), participants highlighted the need for a clearer connection between sick day symptoms and corresponding actions for patients. Suggestions included labelling symptoms, such as diarrhoea, and incorporating explicit images to aid patient understanding. Participants recommended symbols representing symptoms above each tablet to allow patients to associate or link tablets clearly to symptoms.

Consensus among healthcare workers supported the idea of updating tablet images with higher resolution images to differentiate between medications effectively. Participants proposed including images of each tablet within the stop symbol, creating a visual link to indicate whether they should be paused or continued.

Appendix B in Data S1: Themed interview quotes provide a collection of key insights and perspectives categorised by relevant themes, offering a deeper understanding of the qualitative data gathered in this study.

Participants feedback and iterative refinements resulted in a more comprehensive and user‐friendly sick day management plan resource, addressing key concerns raised during the reflexive monitoring process (Figure 1).

## 4 Discussion

This study highlights the desire for tailored patient education resources in rural and remote communities, particularly designed for patients and Aboriginal and Torres Strait Islander health workers. Despite an overall interest in the sick day management resource, there were some clear challenges that impacted successful implementation.

Aboriginal and/or Torres Strait Islander health staff expressed a desire for a resource to support them in educating patients on CKD, AKIs and medications, whereas non‐Indigenous staff were concerned about the perceived complexity of implementation. To address these challenges, this study employed a collaborative approach to designing the resource, involving content simplification, including Aboriginal and Torres Strait Islander artwork and local language, and adopting a flow chart structure to enhance effectiveness. The active involvement of Aboriginal and/or Torres Strait Islander individuals in the design process, including providing images for basic client education, was deemed vital to improve patient engagement and reflect cultural nuances [21]. Safety concerns regarding poor eyesight and health literacy were identified as potential risks leading to tablet misidentification.

The findings of this study emphasise that sick day management resources in this context should prioritise visual elements to minimise the reliance on words. Existing resources for sick day management plans frequently incorporate pictorial representations to illustrate symptoms that indicate the need for plan activation [11, 13, 22]. However, these visual depictions are often accompanied by descriptions that do not reflect the local terminology used in Cape York Peninsula communities (e.g., ‘diarrhoea’ vs. ‘runny tummy’). Additionally, while these resources may include images to signify whether medication should be continued or discontinued during an episode of acute illness, they predominantly rely on extensive written explanations [11, 13, 22]. In contrast, this study's sick day management plan prioritises using pictorial representations to reduce textual content and enhance accessibility. Moreover, current resources do not distinguish between SADMANS and non‐SADMANS medications through written lists or visual depictions—an innovative feature integrated into the present study's management plan.

Pictorial explanations, tablet images and symbols in the sick day management plan improve adherence and patient understanding [23]. The use of images and pictorial explanations has been demonstrated to improve comprehension by 40%–93%, thereby increasing the efficacy of resources [23, 24, 25, 26, 27]. Recommendations include a flow chart structure, Aboriginal and Torres Strait Islander artworks and directions in the native language for improved engagement and information retention.

The study involves implementing a resource in both FIFO specialist services and community‐based clinics, emphasising patient education by the Cape York Kidney Care team and community‐based staff's role in implementing sick day management plans. Encouraging people with CKD to present to community clinics when ill aligns with existing literature on positive patient outcomes through regular engagement with their Aboriginal Community Controlled Health Organisation [28]. This study highlights the need for resources and training to support community staff in delivering chronic disease management education.

The safe use of sick day management plans relies on patients' ability to read and understand the resource. Poor eyesight and limited access to optometry services pose risks of tablet misidentification, potentially leading to AKIs. To mitigate these risks, the study suggests tailoring recommendations based on medication diversity, advising patients without high‐risk medicines to exclude all tablets during illness or encouraging clinic visits.

### 4.1 Strengths and Limitations

The NPT Framework guided question development and thematic analysis, providing an implementation‐focused guideline [29]. Conducting interviews in person in each community allowed contextual insights, where interviewers could note more subtle cues and intonations that might be missed in other formats [30]. The inclusion of staff from diverse health disciplines in multiple communities allowed for data triangulation, enriching the analysis with multiple perspectives on the same topic.

Performing data collection while embedded in communities not only strengthened the researcher's understanding but also fostered rapport with community. This approach meant the data were analysed through the lens of the context and any findings or recommendations would require careful consideration of their applicability in other Aboriginal and/or Torres Strait Islander communities. The variable degree of health literacy among participants impacted the individual's focus on different aspects of the resource, and that may have impacted the overall feedback on the resource's utility Additionally, the language diversity among participants could have contributed to varying perspectives on the resource's relevance.

### 4.2 Future Direction

This research has highlighted some important practice changes that the Cape York Kidney Team have embraced, including regular in‐services at every outreach clinic, to improve cognitive participation in the management of kidney disease, including sick day management. Evaluating effective ways for knowledge sharing between specialist outreach clinics and remote primary healthcare clinics to improve patient outcomes would greatly benefit the integration of care between primary and tertiary services. To progress the use of sick day management plans in this context, the authors recommend a co‐design process with consumers and carers as the next step.

## 5 Conclusion

This study collaboratively designed a sick day management resource for people with CKD, informed by the perspectives of primary healthcare workers in remote Queensland communities. The insights provided by staff guided the development of a resource that prioritises minimal text and emphasises the use of images, ensuring clarity and readability. The incorporation of Aboriginal and Torres Strait Islander artwork aimed to enhance understanding for both healthcare staff and clients. Healthcare workers expressed a need for more educational resources to support their interactions with patients, recognising the potential of a sick day management plan as a valuable aid in their toolbox for patient education and management, addressing both CKD in general and specifically during sick days.

The feasibility of implementing sick day management tools, however, was questionable, and this study underscores the need for further investigation into cost‐effective and time‐efficient methods of generating individualised sick day management plans with up‐to‐date and relevant information. This ongoing research could contribute valuable insights into optimising the implementation and effectiveness of personalised sick day management resources.

## Author Contributions

L.C., A.C., L.B., B.G., A.M. and S.T. conceptualised the research and methodology. L.C., A.C. and B.G. managed data curation. A.M., A.C., S.T. and B.G. managed funding acquisition, project supervision and administration. L.C. and B.G. analysed the data, supported by A.C. and S.T. K.N. provided Indigenous governance and cultural guidance. L.C. wrote the original draft. All authors reviewed, edited and approved the final manuscript.

## Ethics Statement

Ethics for this study were approved by the Far North Queensland Human Research Ethics Committee (HREC/2021/QCH/72719 (May ver2) – 1519).

## Conflicts of Interest

The authors declare no conflicts of interest.

## Supporting information


Data S1.

## Data Availability

The data that supports the findings of this study are available in the Supporting Information of this article.
