# Supplementary material for: Sick Day Management Plans for Aboriginal and/or Torres Strait Islander Peoples With Chronic Kidney Disease on the Cape York Peninsula of Australia: Health Workers' Perspectives
Source: Aust J Rural Health. 2025 Jan 25;33(1):e13223. doi: 10.1111/ajr.13223 (PMC11771647; doi:10.1111/ajr.13223)
Supplement: Supplementary file 1 — Data S1. [file AJR-33-0-s001.docx]

## Supplementary Material

### Appendix A: Interview Schedule

1. What are your current recommendations for patients on managing their medicines during sick days?

Clarifying questions:

- - Are patients recommended to exclude medicines on sick days?
  - How are patients recommended to exclude medicines on sick days?
  - Are patients recommended to exclude specific medicines?
  - How long are patients advised to cease medicines during sick days?
  - When are patients advised to recommence their medicines?

1. How are recommendations on management of medicines during sick days delivered to patients?

Clarifying questions:

- - Do health practitioners utilise tools such as diagrams, anecdotes, verbal explanations etc.
  - If diagrams or physical tools are utilised, do patients get to keep any resources for their own reference?

1. Can you identify any potential barriers to patient-initiated exclusion of SADMANS medicines during sick days?

Clarifying questions:

- - What factors may make it difficult for patients to identify the medications which need to be excluded?
  - Do patients having their medications Webster-packed pose a barrier?
  - Do you believe patients can reliably differentiate between each of their medicines?

Questions 1-3 aim to establish the pre-existing resources and practices in use by the Cape York Peninsula Kidney Team to determine the context of the study. **(NPT- Defining the Context)**

1. Do you believe providing patients with education tools to keep guiding their own medication management would be effective? Why or why not?

Clarifying questions:

- - Would this increase patients’ abilities to know when and how to exclude medicines?
  - Do you believe patients are likely keep information and/or they are provided?
  - Are patients likely to be understand written information provided on education tools?

Question 4 investigates participant opinions on patient cognitive participation in additional pharmacist-led counselling on sick day medication management. ^35^ **(NPT – Cognitive participation).**

1. How do you believe introduction of additional counselling would affect the workload and participation of healthcare workers providing renal care?

Clarifying questions:

- - Are patients likely to remain engaged during consults if additional time is required for counselling on sick day management?
  - Are health practitioners likely to have the time available to invest in additional counselling on sick day medications management?
  - Do you believe health professionals have the knowledge to deliver counselling on sick day medication management?
  - Do you believe health professionals would require additional time for training to gain knowledge for counselling on sick day management?

Question 5 investigates participant opinions on how compatible additional counselling on sick day medication management would be with existing work practices, consult time constraints and the overall goals and activities of the Cape York Kidney Team (collective action). ^35^ **(NPT- Collective action)** This question also aims to establish health practitioner perceptions of the intervention (reflexive monitoring). ^35^ **(NPT – Reflexive monitoring)**

1. Do you believe pharmacist participation in patient counselling on medicine management on sick days would be beneficial?

Clarifying questions:

- - Do you believe pharmacist participation in patient education would alleviate time pressures on health practitioners during consults?
  - Do you believe the medication counselling skills possessed by pharmacists would enable additional patient engagement and information retention?

Question 6 aims to establish the effect that pharmacist involvement would have on the practices of the Cape York Kidney Team **(NPT - collective action)**, the level of patient engagement and information retention **(NPT - cognitive engagement)** and participant opinions and perceptions of additional pharmacist-led patient counselling on sick day medication management **(NPT - reflexive monitoring)**. ^35^

**References:**

1. Murray E, Treweek S, Pope C, et al. Normalisation process theory: A framework for developing, evaluating, and implementing complex interventions. BMC medicine. 2010;8(1):63-63. doi:10.1186/1741-7015-8-63

### Appendix B: Themed interview quotes.

#### Defining the Context.

| "I mean, we sort of attach something similar, not highly similar to that, to their Webster packs and that. But yeah. That’s, yeah." |
| --- |
| "Yeah because I know some patients know when there's a tablet missing from their pack. And some don’t know their tablets or medications." |
| "Yeah, because we already attach a separate sort of letter to their Webster packs for them to come back if they need a review for their Webster packs and that sort of thing. Like as a reminder. Because sometimes when we just give them their Webster pack they don’t really come back." |
| "I know that there’s some that if they feel unwell and they’ve started on a new medication and they feel unwell, they come and tell us and we go, 'this is probably why you are feeling sick.' And then I just refer them to the doctors and they’ll go, 'okay maybe it’s the new medication.'" |
| "So I don’t know if they get any education surrounding that, that I can tell you." |
| "Have you ever talked to the Cape York Kidney Care Team or any renal care specialist about how to manage that sort of thing when you do get a patient in?" |
| "So, yeah, this will help them to give them something to talk about. Because our health workers at at the moment they are Cert 3 going on to Cert 4. They’re all nervous now, because when we say, 'Okay, you guys, need, you know, you can start billing certain items and things like that.' They’re like, 'Well what are we going to talk about? You know, we’re going to start with, you know. You get referrals. Got a 20 min consult. You know you have to, and that’s when you can build." |
| "And so we’ve been talking with Dr. Fiona, and she’s around the chronic disease, and she’s just printed put some stuff, some pamphlets to give to the health works to say, 'okay, when you’re in your consult, from your referral, having a yarn, touching base, here are some things that you can talk about.'" |
| "So you mentioned the brands can get switched for medicines and that sort of thing. Do you see that happen very often? Yeah. Even for myself. For my blood pressure pill, 'Oh no. Are you sure?' Or its, for this tablet we don’t have any 20mg, so you have to take two. So like, you know, I think I’m relatively smart. So when you have someone that you know, one of our older people or someone that hasn’t finished school, and they just, everything is just visual for them. It does throw them. And you get, I mean, my mother was like that. 'You know, these aren’t pills I’m taking.' And then if they start feeling sick, 'Oh poisoning me.' So, you know, that’s just what I witness with my own families and my own relatives. So the brand changing it does throw a lot of the the older boys and girls. So yeah, I mean, what do you do?" |
| "The Webster packs it’s the most problem because they’ve got a little um, concoction going on in each of their little things." |
| "Some of them that are regular medication that they’ve looked at their Webster pack, and it only happened just before Christmas, looked at the Webster pack, shook it up a little bit, said, 'One of the ones that I don’t I don’t know about.' And because we have no doctor here, or no nurses, just health workers, we couldn’t do anything about it." |
| "Yeah, especially, I mean, if they’re on a Webster pack, they know how many is in there and what they look like. Yeah, I don’t know if you know if, you know, if they’ve got 5 pills in there, and they can identify that its these blue ones that are making them ill." |
| "Yeah, because we just spoke with Dr Fiona, she’s a chronic disease doctor, for a long time. The Cardiology Clinic for Cape York. So she was talking, and I said, 'Look, that sort of things, changing brands and this and that, throws them.'" |
| "Some of the Webster packs that I do have, you know, it’s changed. The brand’s changed." |
| "And so they’re gonna be having all these different ones. You know, and so if you’re 80 and your eyesight isn’t good, and you go, 'Well, is that the same as that? I don’t know.' They just can’t tell." |
| "So yeah, so we make it by the, you know, we’re on 100%. You don’t get too many but because that’s how we do it. So we always um, say to people, 'Now, you got your Webster packs? Okay.' And the next day I’ll say, 'You’ve got your 7 day Webster pack?' And then I say, 'Your 14 day one?' And they’ll say, 'Oh, no. I think, yeah, you guys must be run out.' And then I say, 'So have you got all your Webster’s and your medications to last you until then?' And they’ll go, 'Oh no.' And then I say, 'So, can we do you a new one?' And the other one, 'And I’ll pick it up for you because I want it to be done now.' So it’s, you know, and then there is the next one the doctor’s, if they, you know, they’ll put them on a new one. And they’ll come in and I’ll just check with the doctor, and the doctor just lets us know, yeah. And I do a new one, 100% for me, for myself. You can’t do a 7 day and then a 14 day one. So, we’ll always do the 14 day ones. That’s how I manage that." |
| "We have 2 doctors that are fully accredited. You know, to the 100%. And if they’re only doing 7 days, well, the doctors just have to, because they’re not doing them for, you know, that’s just another prescription. But its, for the Webster packs its just gonna, I’m always doing them. And its a week to make up." |
| "You know, the brands can change and it does throw them. Even when they take um, those new tablets to the doctors and they change the brands on them, and they don’t know, but the the person taking them knows. So it throws them. Sometimes for myself. If I change brands. I think, 'Oh God, I’ll take these ones.'" |
| "There was a few there that were asked about that, and the Webster packs, the names of them, the medications, are changing. And they’re just going, 'What is that?'" |
| "Like the GPs don’t do that sort of thing for them. They’re just, 'Yeah, that’s what the tablets are. Take them, because that’s what they’re for. Don’t worry about it.' And they’ve been the same tablets for a long time and yeah, change to a different brand or the form of it. The form is different." |
| "And it’s the same when we first get the Webster packs, and they’ll just say, 'Don’t change the brand.' Because its, you know, its what they, that’s what the doctors have told us to take and then the form, you know, the capsules inside the, yeah. Because we’ve had that sort of things. Its the formula in them. You know, its different." |
| "Well, even my aunties, when they come and visit me, and I’ll go, 'I’ve only got the other one.' They’ll say, 'Oh no, well I’ll take them and see how I go.' And then she’ll bring them back to me, and I go, 'Oh God. Those tablets.' So, but there is a lot of it. And they’ve told the doctor and they’ll just say, 'Oh yeah. Well, don’t take it.' They’ll just say, 'Don’t take them. They just have the other ones.' And then, when they come and visit me. It’s just a lot of changes with them. So, I don’t know." |
| "So, in their Webster packs they can actually see when they, so they know how many pills, and tablets and capsules are in there. And so they can actually, we can actually change, because there’s just little stickers at the front. So, we just stick the stickers to them. So there's the 28 Webster packs for the 28 days for the tablets and things." |
| "Sometimes they, the health workers that are giving them, they’ll have someone and just say to them, 'Do you know what each of the tablets are for?' And they’ll say, 'Yeah, I know what they are for, but I just don’t know what they look like.' And they’ve been telling that since I’ve been in Cairns. I had that happen so many times. Because they’re changing to a different one and its because of the, you know, the problem with that I think." |
| "The things that we have on the electronic dispensary and the AODs and the charts, you know the ones for the patients. And they just look at them. And they know how many they’ve got there. And so they do." |
| "You know, that’s something I say because I always go with this, and you know, I always tell everyone, that’s just the no-brainer, you know. It’s just one of them, if they don’t know and they just, 'Don’t look at them. Do not look at them. Just tell them, 'Don’t look at them.' Because there’s different medications. And so, you know, they, if they go to the doctor, and that’s what they’re on. And the, they’ll just say, 'Yeah, but, you know, the health worker knows and can look at that and tell them that’s what they are.'" |
| "I can get my health workers to print the list out. And I say to them, 'You need to tell them if you want them to know what it is. Print the list out and give it to them so they know what it is and what its for.' Because we are like um, you know, the new medication, a lot of the old people will bring it to the health workers and say, 'The doctor gave it to me, but I don 't know what it is. And I don’t know what it is for.' Because it changes from the tablets, you know, the APO, the Websters, all that stuff. And you know, it does get hard for them because they’re saying, 'The doctor just said to take it, and I don’t know what it is.' But the doctors don’t know what the tablets are. They don’t even have a name on it, just the ones that they’re on. Because the old people get that, and I said, 'You have to print it out for them.' And you know, that’s why I’m saying, 'We have to print it out so they know what it is.' Because the old people bring it to us and say, 'We don’t know what it is.' And I say to them, 'Print it out.' So, um, you know. They’ll say, 'This is for my heart, my sugar and all that. And I know what they’re for.' But yeah. That’s what I say to them." |
| "You know, sometimes they’ll change. And so if they know what they’re for, and they know that the doctor has given it to them, they’ll keep taking them, even if they don’t know. You know, like, they’ll just keep taking them. They don’t know what they’re for. But they’ll just keep taking them." |
| "When I print the list out, I’m saying to them, 'That’s your medication. That’s what you’re taking. And if you’re not gonna give it to them, that’s the one. They don 't know how many they’re taking because there’s two or three in one of them. And they’ll just keep taking them because they don’t know what they are.' Because they just, you know. It can, 'This is for the heart, this is for the sugar, and this one’s for the blood pressure.' And they’ll keep taking them." |
| "The doctors will have to write it on the list for them. And give it to the patients, you know. And so when I write them up a Webster pack for them, I’ll have to put it on there. But if the patients, and the doctors will have to write the list out for them. The doctors, you know, they don’t do that. They’ll just say, 'Just take that.' The doctors, that’s the ones that I, you know, for the hospital." |
| "I do know that because the doctor will have to put it on a list. The patients, they’ll just say, 'I’m on all this medication. I don 't know what it is.' And the doctor just says, 'Just take it.' But the doctors need to put it on a list so they know what the medication is." |
| "You know, even when I have to get my scripts from the doctor, they won’t put it on there. It’s just a lot, you know. And then you’ve got all this medication. And if you don’t know what it is, it’s not gonna help you. They need to, I think they need to put it on a list. And give it to the patient so they know. Because they, they’re just, 'What is this medication for?' And they’ll just say, 'I don’t know. Just take it.' And that’s not, it’s not good. The doctors need to write it on a list for them." |
| "And they, you know, the doctors will say, 'You’re taking that for your heart.' And sometimes the people, and the doctors, they’ll just say to them, 'You’re taking that for your heart.' And then they’ll say, 'I’m taking that for this.' And they’re just not listening. They just, and the doctors will say, 'This one’s for your heart.' And the patients will say, 'I’m taking this for my sugar.' And they’re just not listening to the doctors, you know. The doctors will say, 'This one’s for your blood pressure.' And the patients will say, 'I’m taking this for this.' And so they’re not listening. And it’s gonna be a problem because the doctors have to write it down for them. The doctors need to write it on a list for the patients, and give it to the patients. Because they’re not gonna remember all that. Because the doctors will say, 'I told you what it’s for.' And the doctors have to write it on a list for the patients. Because you just know, sometimes they’re not gonna listen. And they’ll say, 'I don’t know what it’s for.' And then the patients, they’ll just, 'Yeah, I’m taking it for my blood pressure.' And then they’re not taking it, and it’s the heart tablets. And so, you know, the doctors need to write it on a list for them and give it to the patients. So, um, you know, when I write them a list and write them the list out, they’ll just, the doctors will just say to them, 'Just take it.' So the doctors need to write it on the list. So they know what it is." |
| "I think that’s why the doctors, you know, and the patients are saying, 'I’m taking this for my sugar.' The doctors just say, 'I told you it’s for your heart. And they’re not listening. And I say to the doctors, 'You need to write it down for them.' Because they’re just not listening. And they’ll say, 'I don’t know what it’s for.' Because they’re not listening. So the doctors need to write it on a list for them." |
| "Sometimes the doctors, and the doctors will just say, 'I told you that’s for the heart.' And the patients will just say, 'No, I’m taking it for my sugar.' And they’re just not listening. And I think that’s what they do. You know, and then sometimes the doctors, and the patients, they’ll just say, 'I don’t know what this one is for.' And then the doctors will say, 'I told you that’s for your heart.' So I just, you know. So they, I’m saying, 'You need to write it on a list for them.' Because, and that’s gonna help them." |

#### Coherence

| "I think that would be great. Oh, that would simplify a lot of questions they might have but not know how to ask." |
| --- |
| "I think even for me, that would be great for me to go, 'Okay, if you’re taking your perindopril and this makes you feel like that. This is what you need to do.' I think that would be a great resource." |
| "No, I think it’s quite good. My initial thing was, I just look straight down here [referring to the table], but I guess the pictures [the top panel of symptoms] and all that stuff in the background take away from it. It’s just lost. I actually went straight there and then it’s like, 'Okay, if you’ve got a running tummy and vomiting, you know.'" |
| "And without health promotion and things like that to get the community to start thinking about their own health. So like we’ve got resources like that. I like that we’ve got pictures of tablets because all the old boys and girls but not that doesn’t look like my tablet, and it takes about 5, 10 minutes to explain that it’s actually different. The same thing is just a different brand. It’s still toothpaste, it might be one is Colgate and one is Mcleans or one of the others. You know? So, I like the fact that they’ve got the pictures." |
| "I guess if they know how to say it and they see it all the time." |
| "So that’s what I find myself saying because it relates, you know because it was me. So you think, that is good [sick day management plan]. You know." |
| "This is good, but you know, going back to like you’re saying, they might miss, you know, take this tablet or whatever. I don’t know how much easier for that person to take those tablets from the Webster pack. Because when they give them the separate boxes, you know, it’s a bit harder. You know, get mixed up and that." |
| "I would recommend to stick with that, and not have the reason for the medicine." |
| "Like I think medication counseling is great. Knowing what your medication is? What it’s for? And what you’re trying to prevent?" |
| "I think having a sick day management plan should be separate to that because, like just having the name, what it looks like, and to stop what to keep going. Because otherwise, they might get overwhelmed." |
| "What’s vomiting in language, or even dehydration? Like having a bit of an explanation about that." |
| "You pop it out, there are eight tablets in one blister, so it’s really hard to tell them, and a lot of them are white. So you can’t say don’t take these little white ones, take this big one." |
| "So the images, you’d have to have different options sometimes or you’d have to tailor the management plan to the actual generic medication that the patient has. And I have different options to print out, I think." |
| "That’s right. A healthy picture, a sick picture is good to know the difference. To know the difference between the healthy body and the sick body. The kidney is very important. And why the kidney is very important, you know, for living." |
| "All the different brands have different colors." |
| "I don’t know whether it’s safer to just give them the okay for things rather than, you know, don’t take those, do take those. Or just so, you know, you can take vitamins and magnesium rather. Rather than, don’t take that, don’t take that, I don’t take that, only take that, only take that. But again, there’s that confusion of the similar tablets. They’re both white. They’re both round." |
| "I think this could work with a certain subset of clients." |
| "It probably maybe is more complicated potentially than it might need to be just to stop harm, I guess." |
| "You know, if they missed their vitamin D and calcium and magnesium for a day, because it’s easier to say stop all tablets, that might be more straightforward in terms of conveying that. Because the harms of missing those two medicines for two days are probably negligible." |
| "To the harm of having to pop them all out and going, oh this one looks like that one. And like, you know, that could just be a bit of confusion that may not be necessary, or extra work on the patient’s behalf that may be prohibitive or not necessary." |
| "Like a NOAC for someone that’s got Atrial Fibrillation, you might be a little bit more reluctant just to withhold that for a couple to three days. Given that that’s a potential stroke risk, for example, and maybe there should be. Maybe you could focus on, this one might still be important for you to take. And then that messaging gets a little bit complicated and it’d have to be individualized and wouldn’t be something you would be able to put out to everybody." |
| "Maybe something on that like that says, as again as a one size fits all sort of approach, 'go into the clinic if you’re sick, but also make sure that you don’t take your tablets' or something." |
| "You’d want to probably make sure it was consistent messaging across all of the different services." |
| "My only suggestion for how to make it easier for people to understand, I suppose, is people up here respond really well to flow-chart-designed things." |
| "Like you want to see in our clinic, there’s lots of visual cues. Yeah, it’s not written down as a sick day management plan. Using digital cues works much better, especially when English isn’t their first language. It is, but it isn’t. But I think this would work. And it’s something that could be replicated easily." |
| "Explain it. It’s so simple. Anyone could learn it. And then that could be re-taught. You don’t need to be a professional. The professional knows what Prexum, what the actual words are like Spironolactone and vitamin D and things like that. But the other stuff is basic, and you teach someone how to do that as well. So it’s good. I think it’s good." |
| "I suppose, if you had an electronic medical record that someone came in for a medication issue related to renal, you could actually get a bit of data off it. But that’s down the other end of the scale, yeah." |
| "I’d put a picture of the drug." |
| "So, some of the other feedback I’ve also gotten was potentially getting it in the Wik language. Do you think that that would be beneficial for patient engagement? HS6F: Yeah, absolutely, yeah." |
| "Essentially, a lot of the kidney patients have diabetic neuropathy and associated retinal damage, etcetera. And the metformin looks a lot like magnesium and Panadol for that matter. Misidentification is going to be an issue there, too." |
| "So many patients have their medicines Webster Packed. Do you think that would be an issue in taking out potentially nephrotoxic drugs? HS6F: Yep." |
| "Yeah, just explain and make sure they fully understand why they can’t take their tablets because they'll associate kidney failure and the tablets then. If the kidney team tells them not to take it, they’ll have that fear to go back on the tablets again. Maybe not the same one, just any tablet. A tablet is a tablet to them, you know?" |
| "I mean, that’s pretty close those two [referring to the two white tablets that are similar in appearance]. That’ll probably be the only confusing thing here. And this is all pretty." |
| "Yeah. Dehydration. I don’t know if they all know what dehydration is." |
| "Yeah, the main issue you see here is the similarity between those two tablets." |
| "I reckon if you explain to them, what’s the difference? HS7F: Yeah, if Andrea or whoever sits them down, let’s say, 'this one is Metformin. See the number and the shape. This one is magnesium.'" |
| "I think most of our chronic kidney patients are on a Webster Pack, so the issue with that would be understanding what each tablet looks like to pick it out. Most of them are pretty good at taking their tablets from their Webster Pack, but it’s difficult for them to pick out certain tablets." |
| "Most of our chronic kidney patients are on a Webster Pack, so the issue with that would be understanding what each tablet looks like to pick it out. So, while most of them are pretty good at taking their tablets from their Webster Pack, it’s difficult for them to pick out certain tablets." |
| "The issue is whether or not all the medications look the same. Because the brand of the tablet we use changes probably monthly sometimes. So it’s just a continuation of that, but otherwise very handy for them to know what they can and can’t take." |
| "But yeah, is there the ability to create like a template that you can do up with a particular patient's medications? So you can have a drag and drop of, they are actually on all of these and you can add other ones in or take them out." |
| "Well, they all do look exactly the same. Well, go look in the pharmacy. They changed all the bottles so now they’re all in a small little blue bottle. And I’m like, you can’t have three drugs in a row that have three drugs that are completely different but are in exactly the same bottle." |
| "Yeah. Well as I was saying before, like the different brands so different tablets will look different and I guess it depends on what time of day. Like, if they can clearly identify that they take a different little white tablet at a different time of day, or it would depend on what they’re specific pack on how difficult it would be. And how they all have a different level of understanding and ability to manage." |
| "Yeah, unless they know those tablets and that’s pointed out. I mean, yes, the doctor did show them the tablet. So, picture form would be good. Do you have a diarrhea picture? You see how you’ve got your muscles and what they do and stuff? Do you have a diarrhea and vomiting one? You see how you got your muscles, and what they do is that. Do you have one that’s like a diarrhea, one, or vomiting one picture, you know, like something like that. A picture of someone having diarrhea like a cartoon character. Diarrhea or vomiting and then a picture against that tablet." |
| "But they can’t see that. It needs to be here [referring to above the picture of the tablet]. Yeah. Because people relate to, if they can look at them. I don’t know. Oh, you’ve got the picture there, so yeah, I don’t know if that can be bigger so that they can look at the. [Referring to the symptoms panel]." |
| "Because a lot of them are having Webster Packs. So whether they can just pick out the tablets, I guess from there? Yeah. But I think pictures of diarrhea or someone, not there but there in picture form (referring to above the pictures of the tablets) will help. Yep." |
| "Yeah. You see how you’ve got stop. Whichever tablet it was. It was a blood pressure tablet, and you see how you’ve got the muscle symbol. Put them down here. Because a lot of them are having Webster packs. So they can check the tablet in their Webster pack and say, this is the one. A lot of people know their tablets, so that might work, where in urban you would. People might know." |
| "Yeah maybe put a label over here that if you’ve got diarrhea. It’s good you’ve got it at the top. But down here [in the tablet image panel] have a picture if you have diarrhea and then stop." |
| "You know I’m a little bit confused because I thought this was about stopping your medication on a sick day. But I can’t see how it’s related to how it’s going to hurt your kidneys. Unless someone told me that. Yeah, people would relate to that." |

#### Cognitive Participation

| "We could use this to attach to our patients' Webster packs, making it easier for them to understand their medications and ask questions." |
| --- |
| "This would simplify answering their questions and help them when they might not know how to ask." |
| "Patients who come in for Webster packs are generally open to new ideas and information." |
| "I believe patients would be receptive to this, especially since I often receive questions about their medications." |
| "This tool could be beneficial for patients. They can take it home, put it on their wall, and refer to it when they're sick." |
| "If patients are engaging in conversations like this, they would likely be open to listening and learning." |
| "Promoting community health and providing clear resources, like the ones with pictures of tablets, is essential." |
| "How do you think this will work with patients in Napranum?" |
| "I think it will work. I'm a client myself, and understanding the medications, like Metformin, is important, especially when dealing with side effects like diarrhea." |
| "Patients often don't inform doctors of everything they tell health workers." |
| "This sick day management plan can benefit workers and families, but it's essential for families to step up." |
| "I've never seen anything like this in over 30 years. It helps explain the stages of kidney issues and the potential outcomes." |
| "Do you believe patients would be willing to spend extra time discussing their medicines, reminding them what they look like?" |
| "I rarely hear of patients missing their appointments." |
| "Patients want to manage their conditions, and they won't mind spending extra time on it, at least in Mapoon." |
| "Having a separate sick day management plan is crucial. Patients need to understand the name, appearance, and when to stop medications." |
| "Patients can refer to a single sheet during a sick day episode, making it clear and easy to understand." |
| "Ensuring they can distinguish their medications within the box is vital to enable independent medication management." |
| "It's challenging because blister packs contain multiple tablets, and some are similar in appearance, often white." |
| "Different generic brands of medications make it challenging to identify the right ones." |
| "Patients who get their medications from different sources may receive tablets that look different." |
| "It's crucial to explain that these medications won't cure them but will help manage the damage that's already occurred." |
| "Clear communication and understanding are essential." |
| "Knowing what you're taking, why, and how it affects your body is essential. In-services and education on medications are important." |
| "Indigenous Health Workers knowing regular medications and providing support is beneficial." |
| "Some patients may lose the tool if they put it in their pocket." |
| "It needs to be clear and specifically designed for sick day management to grab patients' attention." |
| "Repetition, reminders, and education during clinic visits are helpful." |
| "Patients may need repeated education to fully understand and remember." |
| "Despite providing thorough education, some patients still have trouble remembering their medications." |
| "Understanding what tablets are for is a challenge for some patients." |
| "Patients often understand they have an illness like renal disease, but may not fully grasp the details." |
| "Most patients understand the end-stage of their condition." |
| "Changing the appearance of a medication can lead to confusion, so keeping it consistent is essential." |
| "Simplifying dosing and prescribing is important, given that patients may not take their chronic disease medications." |
| "Introducing it in the Wik language can improve patient engagement." |
| "Educating patients about kidney disease and its impact is valuable, and visual aids like diagrams can help." |
| "Patients may need time to absorb the information and may require repeated education." |
| "Patients may disengage when they want to leave the clinic, and they might not listen to the information provided." |
| "Clinicians may need something to aim for, and this tool can help improve patient engagement." |
| "I thought this was about stopping medication on a sick day, and I don't see how it's related to kidney health." |
| "Patients may understand with proper explanations." |
| "Patients may be engaged and willing to spend extra time." |
| "Most patients are comfortable calling us, and we would support them if they have a plan in place." |

#### Collective Action

| "I think it would be great for me to understand how to handle perindopril if it makes me feel a certain way. It would be a valuable resource." |
| --- |
| "As long as the patient's medication list gets regularly updated, I think it's excellent. I prefer simple, visual information. Keeping it straightforward." |
| "This resource would help me provide patients with clear guidance. They can take it home and refer to it when they feel unwell. It's a valuable tool." |
| "I believe that patients would likely visit us first when they're unwell. I've seen Dr. Fiona identify tablets causing issues and make adjustments. However, it's hard to predict for sure." |
| "I think patients would come to the clinic and say, 'You mentioned that this medication might be causing my sickness. Let's review it together.'" |
| "I strongly believe patients would contact the clinic if they experience symptoms like a runny tummy, nausea, or any acute illness. They wouldn't manage it at home, which would be the compliant approach. Non-compliant patients might just stop taking their medication." |
| "I think it would be beneficial to support health workers during their consultations, which typically last 20 minutes." |
| "Some people have mentioned eyesight issues." |
| "It's up to us. If we print this document on A4 paper, we know some clients have poor eyesight. All we need to do is print it on A3, laminate it, and place it on our desks. We can offer clients a colored copy. Black and white won't be helpful. I don't see any other issues with it." |
| "There's a lack of education for workers about why they're taking certain medications and what to do when they're unwell. It would be valuable for workers to receive this education, particularly in the community." |
| "This resource would be great for HAC staff and support workers, including NDIS support workers, to receive education on." |
| "It's essential to spread community knowledge about medications for kidney disease. People need to know that when they're sick, they should stop taking specific medications. Building community support and sharing this information is crucial." |
| "Using one resource to upskill health workers would positively reinforce the messaging if the team adopts the same resource." |
| "One option could be for patients to call the clinic or visit us. It's essential to help patients when they're sick, and this could be part of a health worker's role. If this approach collaborates with local health organizations like Queensland Health and Apunipima, it would be beneficial. It's also important to ensure that support staff are aware of it." |
| "If there's an option where patients can say, 'I'm feeling unwell, I know I shouldn't take certain medications. Can I talk to you?' It's essential for all healthcare professionals, including nurses, doctors, health workers, and allied health professionals, to be aware of this. They should have access to this information, or have posters on the walls to remind them. When we meet these clients, we can ask, 'Are you feeling unwell? Are you taking specific medications?' We can advise them to stop certain medications while they're sick and document it for follow-up." |
| "Having a brief reminder for patients during their 5-minute appointments or while they're at Queensland Health when they're unwell could be really helpful." |

**Reflexive Monitoring**

| “I like it. Its straightforward and simple for them to understand.” |
| --- |
| “I like resources like that with pictures of tablets because the old boys and girls often say, "That doesn’t look like my tablet," and it can take 5 to 10 minutes to explain that it's actually the same thing, just a different brand.” |
| “I really appreciate that these resources include pictures, as it makes it easier for people to understand.” |
| “Things can only get better with resources like this. In all my years—going back over 30 years—I have never seen anything like this. Resources that explain what your kidneys are going through, the different stages. I think it’s good for our people.” |
| “That would be good cause most of them take Webster packs. So, and if they know what colour their tablet is, and that would be good for them to know which one to take out.” |
| “If they’ve been using it for a long time, they’ll recognize if there’s a small symbol on their table from the pictures.” |
| “I definitely think having Wik incorporated into the educational resources would be beneficial.” |
| I think it would be good if there is an option where they say “I’m feeling a bit sick, I know to not take some of my medications. Can I talk to a health worker about how?” |
| “Having this sort of information available through in-services or by sending it out, or even having posters on their walls, makes a difference.” |
| “When we see these clients, we can point to the materials and say, “Oh, you’re feeling a bit sick. You’re on Webster Packs. Are you taking any of these medications? And then they can learn to manage their own medicines.” |
| “It would be very beneficial for health workers, including Indigenous Health Workers, to know the regular medications someone is on, as well as knowing when they should and shouldn’t be taken. That kind of support would make a significant difference.” |
| “If it’s just a 5-minute reminder each time they come in, it could make a difference. Even having a reminder when they’re at Queensland Health while they’re unwell would be really helpful.” |
| “The thing is, they may associate kidney failure with the tablets themselves. So, if the kidney team advises them not to take a particular medication, they might have a fear of going back on any tablets at all. To them, a tablet is a tablet, regardless of the type.” |
| “I think it’s an age thing. Older patients tend to be more cautious with their medications, while younger ones often have a more relaxed attitude, like, “I didn’t take it today, and that’s alright.” |
| “That’s not really an issue because we already spend quite a bit of time with each patient, going over everything. We do a lot of education around diet, medications, exercise, and lifestyle, among other things. This could easily be incorporated into that routine. Of course, some staff education would be needed, but I don’t see it being a significant problem to add it in.” |
| Most of our patients love calling us, and many have the CNC phone number, so they can reach the CNC directly. We’re always happy to support them. If they have a sick day management plan in place, we would absolutely support them with that.” |
